# Supplementary material for: Are carbon emissions trading and green financial instruments synergistic? -Comprehensive quantitative research based on content analysis
Source: PLoS One. 2024 Mar 7;19(3):e0298601. doi: 10.1371/journal.pone.0298601 (PMC10919601; doi:10.1371/journal.pone.0298601)
Supplement: S1 Data — (ZIP) [file pone.0298601.s001.zip › Data/地级市Z_F工作报告.zip!/║■▒▒/╛ú├┼╩╨/└┤╫╘╣1⁄2╓┌║┼í╛┬φ┐╦╩2╛▌═°í┐_2010.docx]

各位代表：

　　现在，我代表市人民政府，向大会报告政府工作，请予审议，并请各位市政协委员和列席人员提出意见。

　　一、2009年工作回顾

　　2009年是我市进入新世纪以来最为困难的一年，也是我们“在爬坡中提速、在转弯处赶超”取得明显成效的一年。面对国际金融危机的严重冲击和影响，在省委、省政府和市委的坚强领导下，全市上下团结拼搏、迎难而上，全力以赴保增长、保民生、保稳定，在加快荆门崛起的进程中迈出了新的坚实步伐。

　　（一）综合实力不断提升。初步核算，全市地区生产总值达到600.5亿元，增长14.8%，创12年来最高增幅；规模工业增加值259.3亿元，增长27.5%；全社会固定资产投资317.2亿元，增长51%，创16年来新高；社会消费品零售总额228.8亿元，增长21.2%；出口总额1.7亿美元，下降17.4%，实际利用外资1.4亿美元，增长16.7%；财政总收入60.3亿元，地方一般预算收入18.1亿元，可比增长23.4%；单位生产总值综合能耗下降5.5%，化学需氧量和二氧化硫排放量分别下降2.0%、0.8%；城镇居民人均可支配收入13857元，增长9.2%，农民人均纯收入5956元，增长11.7%；中心城区居民人均消费性支出10595元，增长6.9%；城镇新增就业人数4.18万人，城镇登记失业率4.1%；人口自然增长率3.76‰；居民消费价格上涨0.1%。除外贸出口、城镇居民人均可支配收入外，主要经济指标实现了“两高一进位”的目标。

　　（二）发展后劲明显增强。全年施工项目1289个，在建亿元以上项目160个；争取四批新增中央投资项目455个、投资21.7亿元；储备项目2700个，投资总规模6300亿元。李宁加工园一期、中集（宏图）能源装备一期、天茂20万吨二甲醚、众和20万锭紧密纺、汇源果蔬饮料食品加工、凯迪生物质发电一期、洪森天利30万吨油脂加工、爱斯曼食品等项目竣工投产，李宁加工园二期、星球商业中心等项目进展顺利，国家大型森林灭火/水上救援飞机研制项目正式启动，华能热电联产项目获国家能源局同意开展前期工作，荆门核电站项目通过预可研审查，历时多年的国家大型商品粮基地项目获国家批复并下达投资计划。招商引资取得新进展，引进市外境内资金135.4亿元，增长39.5%。全民创业深入推进，新增各类市场主体1.93万户。全市县域经济继续保持速度加快、活力增强、结构优化、科技进步的良好发展态势，完成生产总值362亿元，增长15.6%，占全市经济总量的60.3%，对全市经济增长的贡献率为66.1%。省委、省政府在荆召开现场会，推广我市县域经济发展模式。

　　（三）结构调整步伐加快。三次产业结构由上年的24.8：39.8：35.4调整为22.4：43.2：34.4。石化、磷化、食品和建材、机电、轻纺、新能源等七大产业产值达到841.3亿元，高新技术产业增加值达到33.94亿元，增长13.6%。规模工业企业实现利润39.1亿元，比上年扭亏增盈41.5亿元。荆门经济开发区和各县市经济开发区、十强工业重镇、207国道工业走廊发展势头强劲。企业规模不断壮大，全年新增规模以上工业企业223家，达到1007家,新增亿元企业35家，达到127家，荆门石化等5家企业进入全省百强企业行列，新洋丰肥业跻身中国民营企业500强。现代农业建设扎实推进，粮食总产再次超过50亿斤；农产品加工产值237亿元，增长49.7%；市级以上龙头企业新增17家，达到152家；农民专业合作社新增923家，达到1180家，居全省首位；“三品”标志产品达到315个，国宝桥米、广源食品被评为中国驰名商标；杨竹流域生态文明新农村示范区等试点示范建设全面展开；农业基础设施进一步加强，农机综合作业率达到65%。以文化旅游业为龙头的现代服务业加快发展。全年接待国内外游客663.6万人次，实现旅游总收入30.3亿元，分别增长21.9%和38.4%。京山三阳----屈家岭旅游大环线开工建设，绿林山风景区被批准为国家4A级景区，三泉村等5个旅游名村进入全省旅游名村创建行列。组建了生态文化旅游（集团）开发有限公司和旭冉国际旅行社。房地产、金融、保险、商贸、物流等产业快速成长。

　　（四）循环经济取得突破。编制完成磷化、石化和电子废弃物循环经济产业园发展规划。全年开工建设循环经济项目160个，完成投资111亿元。全市综合利用各类废弃资源企业达百家。工业固体废弃物综合利用率达到99%、工业废水排放达标率90%以上。组织申报国家、省循环经济项目60个，获批专项资金1.33亿元。节能减排和环境保护取得积极成效，夏家湾污水处理厂二期、京山污水处理厂和荆门第二垃圾处理场建成投入使用。国家现代林业示范市建设深入推进，初步通过全国绿化模范城市检查验收，全国首家现代林业森工科技产业园在东宝区授牌成立，漳河水库入围国家湿地公园，城郊已关闭采石场植被恢复工作全面启动。

　　（五）城市建设全面推进。城市规划体系逐步完善，中心城区控制性详规编制覆盖率提高到70%。基础设施建设快速推进，投资6.8亿元，新建改造城市道路79万平方米、绿化40万平方米，城区主干道基本刷黑，主要街道实现亮化，出入口环境明显改善，生态运动公园、凤凰湖公园、象山风景区建设顺利实施，李宁（荆门）产业园、福耀玻璃、中航运输装备产业园等园区配套市政设施不断完善。城市管理力度进一步加大，城区个人建房集中整治取得阶段性成果。钟祥、京山、沙洋3个县城和小城镇建设步伐加快。完成国道、省道大修92.4公里，改造县乡公路60公里，武荆高速公路荆门段已建成，全市二级公路收费站全部停止收费并撤除站点设施，提前一年完成“十一五”交通规划目标。

　　（六）社会事业协调发展。创建创业型城市扎实开展，成功举办全省市县区长就业论坛。社会保障水平不断提高，参保人数达到160万人次，城镇居民基本医疗保险、新型农村合作医疗保险参保率分别达到91%、96%，城乡低保对象达到15.5万人。钟祥列入全国新型农村社会养老保险试点，掇刀城乡居民基本养老保险工作稳步实施，被辞退民师等特殊群体养老保险工作顺利推进。科技创新取得新成绩，第五次荣获“全国科技进步先进市”称号。全面启动义务教育均衡发展行动计划，落实义务教育经费保障机制改革资金1.56亿元，全市义务教育学校教师绩效工资基本落实到位，改造农村初中校舍1.21万平方米。庆祝国庆60周年系列文化活动丰富多彩。京山、钟祥入选全国文化先进县（市）。医药卫生体制改革启动，城乡公共卫生服务体系进一步健全，中心城区“十分钟社区卫生服务圈”基本形成。依法、科学、有序防控甲型H1N1流感，疾病预防控制工作取得新成效。低生育水平进一步稳定，“婚育新风进万家示范市”创建工作通过国家验收。第十三届省运会筹备工作稳步推进。社区“三基”行动计划全面实施。社会治安综合治理扎实有效，严打整治的针对性和社会治安防控的有效性进一步增强。5个县（市区）均被评为湖北省平安县，沙洋被评为全国平安县。“五五”普法广泛开展。信访秩序不断规范，大信访格局初步形成，我市化解信访积案工作的经验在全国、全省推广。安全生产日常监管不断加强，产品质量、食品安全等专项整治继续推进。国防教育、双拥工作和民兵预备役建设取得新成绩。屈家岭南水北调移民安置试点工作在全省率先完成，抗震救灾对口支援任务提前一年完成，援疆工作成效明显。统计、物价、工商、烟草、外事侨务、台湾事务、仲裁、气象、人防、防震、地方志、档案、知识产权、应急管理、扶贫开发、住房公积金管理等工作得到加强，民族宗教、广播电影电视、新闻出版、工会、青年、妇女、儿童、老龄、残疾人等事业取得较大进步。

　　（七）民生问题持续改善。市政府承诺的十件实事全面完成，一些关乎民生的问题得到了较好解决。全年新增城镇就业41848人，组织农村劳动力培训转移51848人，发放小额担保贷款7621万元，扶持2924人成功创业，带动就业11953人；建成廉租住房1667套、8.68万平方米，对城市无房或人均住房建筑面积11平方米以下的14063户低收入家庭，发放廉租住房补贴1835万元；投资500万元，完成28条小街小巷改造，夏家湾污水处理厂二期和杨树港污水处理厂分别完成投资4200万元、4250万元，中心城区环境空气质量达到国家二级标准以上的天数为90.7%；投资1.16亿元，解决了24.95万人的饮水安全问题，新建户用沼气池40039口；建成通村公路1824.5公里、通组公路碎石路面1000公里、路基2000公里，建设候车棚85个、招呼站150个；建成43个村文化活动中心和文化中心户、122个农民体育健身工程、538家“农家书屋”和405家农家店；按每生3000元标准资助了107名大学贫困新生入学，筹集1236万元资助了18106名中等职业学校一、二年级困难学生，筹集38.4万元为4054名城市家庭经济困难学生免费提供了教科书，筹集35.3万元免除了市特校在校学生的学杂费、课本费和生活费；市惠泉学校正式挂牌，综合性医院主体楼已封顶；中心城区1个街道社区服务中心即将建成，6个社区基础设施建设已经完工，新增办公用房和服务用房面积2800平方米；办理法律援助案件860件、其他法律援助事项8600件，为困难群众和农民工挽回经济损失850多万元。

　　（八）自身建设得到加强。认真开展深入学习实践科学发展观活动，扎实开展“能力建设年”、“作风建设年”活动，政府公信力、执行力和约束力进一步增强。坚持依法行政，主动接受市人大及其常委会的法律监督，自觉接受市政协的民主监督，加强与各民主党派、工商联和无党派人士的联系沟通。办理建议135件、提案308件，办理质量和效率不断提高。深化行政审批制度改革，审批平均提速20%以上。认真开展届中审计和执行财经纪律的“大学习、大排查、大整改”活动，建立公务车标识、公务消费公务卡结算、公务接待费用定期公示等制度。加强对中央、省投资项目的监督检查和跟踪审计，成立财政投资评审中心，深入开展“小金库”专项治理，廉政建设和反腐败工作取得新成效。

　　各位代表，过去一年，我市经济社会发展的成绩和进步，是在严峻复杂的形势下取得的，极其不易。回顾一年来的工作，我们牢牢把握了以下几方面：

　　一是始终把提振信心作为应对危机的动力之源。面对危机和困难，市委、市政府把中央、省一揽子政策与荆门实际紧密结合，及时出台扶持企业经营发展的政策意见，大力推行市级领导包联和工作专班服务重点项目、骨干企业“直通车”服务、企业点名服务等制度；各县市区在差异中求发展、在竞争中壮实力，成为保增长的中坚力量；市直部门牢固树立“产业第一、发展为大”的意识，广泛开展“发展竞赛”、“服务竞赛”和“服务对象评科长”活动，共为企业落实税费减、免、退政策6.52亿元，筹集3.1亿元支持重点工业项目建设，向省担保公司和市中小企业担保公司各注资1000万元，帮助重点企业节省铁路运输费用近4000万元、减少用电成本近亿元；广大企业强化管理降成本、克难奋进拓市场，迎来了转机。全市上下凝神聚力、逆势而上、稳扎稳打，奋力实现了年初的“开门红”、年中的“硬过半”、年底的“两高一进位”，掌握了发展主动权。

　　二是始终把大抓项目、抓大项目作为保增长的关键之举。坚持抢字当头、干字为先，深入开展“项目建设年”活动。成立扩大投资工作专班，调整市级招商体制，先后与近10个省直部门签订合作协议，投资规模和增幅双双创历史新高。加强银政、银企合作，分别与省四大国有商业银行签订三年投放405亿元信贷资金的战略合作协议，金融机构新增贷款增长29%，达到60.04亿元。着力优化投资环境，我市第7次被评为“湖北省A级金融信用市”，入选10个“浙商最佳投资城市”和30个“苏商投资首选城市”。

　　三是始终把转变发展方式作为推动发展的根本之策。坚持应急和谋远结合、增长与调整并举，大力实施工业“3+4”、农业“212”、文化旅游“133”计划，不失时机地推动经济结构转型升级。着眼于在保增长中加快淘汰落后产能、在发展循环经济中拓展环境容量、在绿色发展中提高经济增长的质量和效益，成功举办中国.荆门“循环经济论坛”和“生态论坛”，坚定不移“培育大企业、建设大园区、发展大产业、推动大循环”，进一步提高了发展质量，拓展了发展空间，增添了发展后劲。

　　四是始终把共建共享发展成果作为政府工作的出发点和落脚点。越是困难时期，越要重视民生和稳定。我们坚持把保增长与保民生、保稳定统一起来，着力办好市政府承诺的十件实事，积极落实深入学习实践科学发展观活动整改事项，努力“察民情、排民忧、解民难”，较好解决了一批关系民生的实际困难，解决了一批影响稳定的突出问题，保持和维护了社会和谐稳定大局。

　　各位代表，这些成绩的取得，得益于省委、省政府和市委的正确领导，得益于市人大、市政协的有效监督和大力支持，得益于全市上下的同舟共济、共克时艰。在此，我代表市人民政府，向全市人民，向驻荆部队、武警官兵、政法干警，向各民主党派、工商联、无党派人士、各人民团体，向长期关心支持荆门发展的各界朋友表示衷心的感谢，并致以崇高的敬意！

　　在充分肯定成绩的同时，我们也清醒地认识到，政府工作与全市人民的期待还有不小差距，前进中还面临不少困难和问题。主要是：经济总量不大，整体实力不强；重大产业项目不多，主导产业集群发展不够，产业链条不长，产品科技含量较低；资源环境约束加剧，节能减排任务艰巨；城乡一体化工作刚刚起步,农村基础设施还比较薄弱；民生保障压力加大，就业、医疗、住房、交通等与群众生活密切相关的问题亟需加快解决；政府职能转变还不到位，少数工作人员的思想观念、作风和能力还不完全适应科学发展的要求，等等。对于这些问题，我们将采取更加有效的措施，认真加以解决。

　　二、2010年的主要任务

　　2010年是执行“十一五”规划的收尾年，也是谋划“十二五”发展的基础年。做好今年的政府工作，至关重要。

　　今年政府工作的总体要求是：深入贯彻落实党的十七大、十七届三中、四中全会和中央、省、市经济工作会议精神，坚持以科学发展观为指导，牢牢把握“在发展中促转变、在转变中谋发展”的主线，坚定不移扩总量、调结构、转方式、促创新、惠民生，扎实推进新型工业强市、现代农业强市、文化旅游强市和生态宜居城市建设，努力办好一届高水平、有特色的省运会，全面完成“十一五”经济社会发展目标，为“十二五”乃至更长时期的发展打下坚实基础。

　　全市经济社会发展的主要预期目标是：生产总值增长12%，规模以上工业增加值增长22%，单位生产总值综合能耗下降4.8%，化学需氧量排放下降1%，二氧化硫排放与2009年持平，全社会固定资产投资增长35%，社会消费品零售总额增长20%，实际利用外资增长12%，外贸出口增长15%，地方一般预算收入增长14.4%，城镇居民人均可支配收入增长8%，农民人均纯收入增长7%，居民消费价格涨幅控制在3%左右，新增城镇就业人员4.1万人，城镇登记失业率控制在4.3%以内，人口自然增长率控制在4‰以内。在实际工作中，我们要朝更高的目标努力，力争更好更快地发展。

　　实现上述目标，要坚持以下指导原则：

　　第一，加快发展扩总量。发展不够仍然是现阶段荆门最大的实际。面对新一轮竞争，惟有加快发展，才能赢得政策支持、赢得竞争优势、赢得新的发展空间。当前，我市面临全省实施“两圈一带”、汉江流域综合开发、“宜荆荆”（宜昌、荆州、荆门）城市群建设、江汉航运圈建设等战略带来的重大机遇，有加快发展的条件；我市的经济发展持续向好，进位赶超的态势正在形成，有加快发展的基础；我市的产业和企业扩张力、带动力、影响力不断增强，有加快发展的实力；我市的干部队伍和企业家在危机中接受了洗礼，提高了应对复杂局面的能力，有加快发展的保障。我们要牢牢把握加快发展主题，真抓实干，乘势而上，奋力向经济总量“千亿俱乐部”迈进。

　　第二，调整结构转方式。发展质量还不够高仍然是现阶段荆门最现实的矛盾。面对后危机时代与区域竞争的双重压力，面对转变发展方式与扩张经济总量的双重任务，面对资源短缺与环境容量的双重制约，我们要高举生态荆门旗帜，大力发展循环经济和低碳经济，以发展方式之变应对外部环境之变，以产业升级推动经济转型，变发展潜力为发展能力，变后发优势为竞争优势，努力推动科学发展上水平。

　　第三，统筹兼顾促和谐。发展还不够协调仍然是现阶段荆门最突出的问题。我们既要注重突破性发展城区经济，又要注重做大做强县域经济，促进市域经济整体提升；既要注重提高城镇化水平，又要注重加快新农村建设步伐，促进农村与城市和谐共进；既要注重“好”字当头，又要注重“快”字优先，促进人与自然和谐相处；既要注重扩张经济总量，又要注重最大限度地让发展成果普惠人民群众，不断提高全市人民的幸福指数。

　　为此，我们要集中全力抓好以下工作：

　　（一）强力推进项目建设，保持投资较快增长

　　狠抓先进制造业项目建设。计划新建或续建80个亿元以上项目，完成投资120亿元。重点开工荆门石化装置技改和产品深加工、大峪口公司磷复肥技改、李宁材料工业园一期等项目，推进格林美电子废弃物循环产业工程、京山轻机20万吨精密铸件、沙洋纺织工业孵化园、钟祥金鹰24万吨氨醇等项目，建成李宁加工园二期、宝源木业年产20万立方米定向刨花板、屈家岭福美来30万吨油脂加工等项目。继续抓好中央新增投资项目的续建和收尾，力争尽早建成并发挥效益。加快推进荆门石化扩能改造、国电长源荆门热电厂四期、荆门核电站建设等项目挤进国家、省相关规划“笼子”。

　　加快现代服务业项目建设。计划新建或续建50个亿元以上项目，完成投资47亿元。重点抓好明文化主题公园、京山温泉生态体育公园、绿林景区整体开发、喜民园生态农业休闲观光园、漳河湿地公园、帝豪五星级酒店等文化旅游项目，加快推进李宁物流园、仪邦农贸物流城、人民电器综合物流园区、星球商业中心、荆门粮食物流中心、九禾化工产品物流园、沙洋中心港区物流园等项目。

　　抓好基础设施项目建设。计划新建或续建30个亿元以上项目，完成投资41亿元。交通方面，争取长荆、荆沙铁路电气化改造提速，服务引江济汉工程和荆州--沙洋--武汉千吨级航道圈建设，建好武荆高速公路到郢中连接线，推进荆新一级公路改造，开工建设荆门城区西北环大通道，完成国省道、县乡道改造300公里以上。能源方面，开工建设华能热电联产，开工或续建城东220kV输变电等8个电网建设项目。城市建设方面，拉通漳河大道、象山大道南段、长坂坡路等主干道路，新建凤凰大道、李宁大道、创业四路、龙井大道等道路，配套完善荆门经济开发区市政基础设施。

　　加强农业水利项目建设。计划新建或续建25个亿元以上项目，完成投资21亿元。启动国家大型商品粮基地建设，整治汉江崩岸险情，对杨树土当等30座中小型水库进行除险加固。实施“双低改造工程”，改造建设5万亩高产农田和低丘岗地。

　　推进社会发展事业项目建设。计划新建或续建4个亿元以上项目，完成投资6.4亿元。全力推进荆门生态运动公园及配套设施工程，确保如期交付使用。做好荆门博物馆迁建、市图书馆扩建前期工作。

　　确保项目建设顺利实施。着眼于“十二五”规划启动实施，全力争取国家和省的支持，扎实做好项目的可研和规划编制、环评、申报及跟踪衔接等工作。多渠道筹集项目建设资金，鼓励和支持非公有资本进入基础设施、公共事业及其他行业和领域。继续实行“二分之一”工作法和“两个小分队”服务法，构建项目建设绿色通道，有效解决建设用地、征地拆迁等问题，促进投资快速增长。

　　（二）加大结构调整力度，提高工业经济整体素质

　　做大做强优势产业集群。以实施“3+4”计划为主线，把调整升级任务落实到产业、企业和产品上来，壮大龙头企业，拉长产业链条，促进集群发展，力争石化、磷化、食品和机电、建材、轻纺、新能源七大产业群主营业务收入达到1000亿元。整合资源，强化服务，引导企业在技术创新、产品升级和实行现代管理上狠下功夫，着力在“二次创业”、裂变扩张上迈出新步伐，提高企业的核心竞争力。力争荆门石化销售收入突破210亿元，新洋丰销售收入达到70亿元，国宝桥米集团销售收入达到30亿元，大峪口、天茂集团、中粮祥瑞、洪森集团等企业销售收入超过10亿元，亿元企业新增20家以上，规模工业企业新增200家以上。

　　大力发展园区经济。坚持“布局集中、产业集群、用地集约”的原则，科学编制新一轮开发区发展总体规划，加快完善园区水、电、路等配套设施，进一步提升园区项目承载力。荆门经济开发区要按照“一区多园”的思路，实施“项目满园工程”，大力发展体育用品、汽车装备制造、石化深加工、电子信息等产业，力争固定资产投资、规模以上工业增加值、入库税金翻一番。按照“突出专业、培育品牌”的要求，加快荆门石化循环产业园、李宁（荆门）产业园、航空科技产业园、东宝森工科技产业园、屈家岭现代农业示范区和荆襄磷化循环产业园等特色园区建设，支持各县市经济开发区加快开放开发步伐，力争新增1--2家年主营业务收入突破100亿元的园区。

　　切实增强科技创新能力。坚持政、产、学、研、建“五位一体”，引导企业加大技术创新投入，建立省级工程中心、研究中心、重点实验室和博士后科研工作站，力争新增2--3家省创新型试点企业。以工业园区为载体，整合技术资源，抓好荆门高新技术产业创业中心和聚盛国际SBI产权、格林美电子废弃物资源化等孵化平台建设。力争全年实施工业技改项目37个，完成技改投资45亿元。积极发展新材料、新能源、电子信息、医药化工、通用航空等战略性新兴产业，力争高新技术产业增加值增长15%以上。大力开展品牌质量建设和“名优产品生产基地”建设，推进质量振兴活动，培育精品名牌。深入实施人才强市战略，全面落实我市做好企业高层次人才服务工作的十条意见，鼓励科技人员带头创业，培养和引进一批现代产业领军人才和高素质技能型人才，以人才结构的转型优化促进发展方式的转变。

　　全力扶持企业发展。加大财税金融支持力度，综合运用贷款贴息、以奖代补、投资参股等手段，支持企业发展和项目建设。推进银政、银企合作，加大对中小企业贷款力度，力争全年新增贷款不低于60亿元。积极服务和支持中省企业发展。推进凯龙、新洋丰、宝源等企业继续做好上市融资前期工作，力争1家成功上市。支持新洋丰、凯龙、金龙泉等有条件的企业，采取兼并联合、资本重组的方式，实行低成本收购、低成本扩张。加强工业经济预测分析和运行调节，切实抓好煤、电、油、运、气等生产要素的组织协调，促进企业最大限度释放产能、达产达效。

　　（三）扎实做好“三农”工作，大力发展县域经济

　　深入推进产业化经营。围绕粮食、棉花、油料、畜禽、水产、果蔬、森工、生物质能源八大优势产业，按照“四个一批”的要求，大力实施“212”计划，推动农业产业化向高端发展。巩固和扩大国宝桥米“一袋米”工程成果，鼓励中粮祥瑞、洪森天利打造“双低”菜籽油知名品牌，支持广源、楚玉、爱斯曼等企业发展，力争全市农产品加工产值与农业总产值比达到1.2：1。抓紧实施国家大型商品粮基地建设项目，开展优质农产品高产创建，加快建设优质稻基地200万亩、双低油菜基地200万亩、优质棉花基地50万亩。推广“150”标准化养猪、“1235”养羊、秸秆养牛等高效养殖模式，实施稻渔共生、畜渔配套、良种繁育工程，力争畜牧、水产产值占农业总产值的比重达到55%。围绕龙头企业广泛建立合作经济组织，规范合作社运作，力争全市30%的农户加入农民专业合作社，带动农户达到50%以上。加强农产品品牌创建，力争新认证农业“三品”30个。健全农业技术推广、农产品质量安全、农业生态安全和动植物疫情防控体系。进一步提高农业机械化装备水平，力争农机综合作业率达到68%。

　　努力增加农民收入。积极发展农村二、三产业，稳步发展休闲农业、观光农业，让农民从产业化、城镇化、社会化和机械化中增收。全面落实中央惠农政策，按期足额发放各项农业直补资金，确保农民政策性增收。强化农民负担监测和涉农收费监管。加强农村集体资金、资产、资源管理，逐步盘活村级集体闲置资产，积极化解村级债务，力争60%的村集体经济年收入过5万元，让农民从发展集体经济中得到实惠。继续实施农村劳动力转移培训“阳光工程”，力争全年转移农村劳动力5万人，不断提高工资性收入占农民收入的比重。

　　扎实推进新农村建设。以产业发展、交通建设、高产农田建设、河道整治、水生态修复、造林绿化、公共设施建设和村庄环境整治等八大工程为重点，加快杨竹流域生态文明新农村示范区建设。抓好省、市新农村建设试点镇和示范村建设，推动新农村建设由点到面有序健康发展。积极推进农村安全饮水工程。启动新一轮农村电网改造，改善农村交通、通讯条件。推进“一建三改”和绿色家园工程建设，新建户用沼气池2万口以上，鼓励有条件的地方修建大中型沼气池。推广钟祥彭墩村经验，通过开展迁村腾地，因地制宜推进新农村居民点建设。实施乡村清洁工程，抓好农村环境综合整治，加强农村面源污染治理，不断改善农村面貌。引导社会资本参与农村产业发展和公共设施建设，鼓励社会力量与乡村结对帮扶。坚持开发式扶贫方针，加大劳动力转移培训、产业化投资、以工代赈等各项扶贫工作力度，加快基础设施建设和社会事业发展。继续加大对革命老区、少数民族地区发展的支持力度。完成省政府下达的南水北调移民安置任务。

　　增强县域经济综合实力。全面落实支持县域经济发展的政策措施，推动各县（市区）发挥优势、竞相发展，不断提升在全省整体排名中的位次。以争创“中部百强县”为主题，支持钟祥做大做强磷化产业、农产品加工、旅游等产业；支持京山积极融入武汉城市圈，加快发展机械制造、粮食工业、家禽产业加工和生态旅游业。支持沙洋抢抓引江济汉工程建设机遇，培育壮大轻纺、水产品加工、医药化工等产业，建设汉江明珠城市。按照“市区一体、区为基础”的要求，推进东宝进一步发展壮大磷化和建材产业集群，推进掇刀突破性发展现代服务业和观光农业，推进屈家岭加快建设现代农业示范区。坚持在规划引导、基础建设和资源配置等方面重点支持，进一步下放管理权限，促进工业重镇加快发展，推动207国道工业走廊集约集群发展，力争工业增加值增长40%以上。

　　（四）加快发展第三产业，努力拓展城乡消费

　　加快发展文化旅游业。大力实施“133计划”，努力打造鄂西生态文化旅游圈节点城市和重要休闲旅游目的地。加快旅游基础设施建设，开通京山三阳----屈家岭旅游大环线，积极规划筹建大洪山旅游公路。加大景区建设力度，深化旅游创建，启动绿林山景区、明文化主题公园5A级和漳河景区、象山景区4A级创建工作，支持京山争创湖北旅游强县、打造“中国网球之乡”，鼓励钟祥市彭墩村争创全国农业旅游示范点，力争新增1家湖北旅游名镇、100家农家乐进入全省重点扶持行列。启动纪山楚文化旅游开发。开展“旅游服务质量提升年”活动，强化旅游促销，拓展旅游市场。

　　积极发展现代物流业。抓紧制定物流业发展规划，研究制定支持物流业加快发展的政策措施。加快建设一批铁路专用装货点、专用码头、专用货用支线等物流基础设施。整合提升传统物流，积极发展第三方、第四方物流，引进培育一批具有较强竞争力的现代物流企业。积极吸引国内外企业在荆门设立地区总部、销售中心、结算中心和研发中心，争取更多的国内外中介机构落户荆门。

　　稳步发展房地产业。落实国家房地产调控政策，鼓励居民自住和改善型住房消费，增加中低价位、中小户型普通商品住房供应。加快规模大、标准高的居民小区建设，提升居住档次。以廉租房建设、棚户区改造、农村教师周转房、农村危房改造为重点，加大保障性安居工程建设力度。推动建材下乡，鼓励农民依法依规建设自用住房。规范发展住房二级市场，盘活住房租赁市场。整顿规范房地产市场，抑制投机性购房，遏制房价过快上涨，保持市场房价基本稳定。

　　大力发展现代金融业。充分发挥金融配置资源、调节经济、服务发展的功能，创新金融组织体系和发展模式，创新金融产品和服务。争取引进2家以上股份制商业银行，组建1家农村合作银行、2家村镇银行，支持农总行在钟祥开展“三农”事业部制改革试点，支持钟祥、京山、沙洋3家县级农村信用联社改制为农村合作银行或农村商业银行。鼓励涉农金融机构在乡镇增设网点，推动金融机构、人员、业务“三下乡”。支持优质担保公司做大做强。鼓励企业发行债券，创新城市基础设施建设融资方式，拓宽融资渠道。规范保险业经营秩序，引导保险机构发展涉农保险业务。

　　着力扩大消费需求。加快“万村千乡市场工程”、“农超对接”、“新网工程”等项目建设，支持东方百货等骨干商贸企业做大做强，落实“家电下乡”、“农机下乡”和“汽车、摩托车下乡”补贴政策，扩大农村消费。继续实施“网点进社区、服务进家庭”工程，完善便民商贸服务设施，落实汽车、家电“以旧换新”政策，促进耐用品消费升级换代，扩大城市消费。着力发展服务消费和旅游消费，鼓励扩大信息服务、文化娱乐、体育健身等消费，大力培育新的消费热点，推动消费结构优化升级。改善消费环境，发展消费信贷，保护消费者权益。

　　（五）加强城镇规划建设管理，办好第十三届省运会

　　力促中心城区建设管理上水平。编制完成城市空间发展战略规划、荆门经济开发区总体规划、西城区控制性详规和各类专项规划，完善规划体系。大力实施绿化、亮化工程，建成生态运动公园、象山风景区、凤凰湖公园、李宁生态公园等城市绿化精品，开展5条景观大道后续整治，新建改造22条道路绿化，塑造高速公路出入口精品景观带，争创国家园林城市。抓好给排水、公厕、停车场、集贸市场等公用设施建设，完善城市功能。继续开展中心城区个人建房整治，严厉查处违法建设行为。推行精细化和网格化管理，逐步建立全天候、无死角、广覆盖的城市管理格局。对城市出入口、城乡结合部、城中村、居民小区、菜市场等薄弱环节进行重点治理，着力解决“脏、乱、差”等突出问题。整顿交通秩序，调整优化公交线路，抓紧解决重点地段的交通拥堵问题。

　　推动统筹城乡发展上水平。深入实施“1311”战略，切实加强县城和建制镇建设，加快推进城乡一体化。把县城、小城镇、居民点建设与市场建设、产业集聚发展结合起来，大力发展一批具有产业基础和文化特色的重点镇，引导人口向城镇集中、工业向园区集中、居民向住宅小区集中。完善财税、投融资等配套政策，探索建立多元化的小城镇投融资体制，加大基础设施建设力度，促进城镇化与新农村建设良性互动。抓紧开展城镇户籍制度改革试点，放宽小城镇落户条件，逐步实现农民工在子女就学、公共卫生、住房购房以及社会保障等方面与城镇居民享有同等待遇，推进在城镇稳定就业和居住的农民有序转变为城镇居民。支持掇刀创建全省城乡一体化发展改革试验区。

　　促进文明创建上水平。把承办省运会与提升荆门文明城市的水平和内涵结合起来，加强市民教育，大力开展“迎省运、讲文明、树新风”等群众性文明创建活动，培育良好的社会风尚。加强公共卫生、公共秩序等领域文明行为规范教育，提高窗口单位服务水平。加强社会公德、职业道德和家庭美德建设，培育社会主义核心价值体系。广泛开展全民健身活动。动员全市人民参与省运会，当好东道主，展示荆门文明形象。

　　确保赛会组织服务上水平。把承办第十三届省运会作为事关全局的重大责任，举全市之力，紧紧围绕“激情省运.魅力荆门”主题，按照“一办三促”的要求和特色鲜明、文明精彩、低碳环保、安全节俭的原则，细化工作方案，严格实行目标责任制，精心做好竞赛组织、对外宣传、大型活动、服务接待、志愿服务、安全保卫等工作，以一流的精神风貌、一流的城市形象、一流的组织服务、一流的竞赛成绩，办一届高水平、有特色的体育盛会。

　　（六）积极发展低碳经济，建设生态荆门

　　大力发展循环经济。加快石化、磷化、电子废弃物三大循环产业园建设，力争格林美循环产业园首期建成投产，荆门再生资源利用中心开工建设。抓好企业内部废渣、废水、废气、余热、余压的综合利用和监督管理，加快葛洲坝2&times;9兆瓦余热发电、荆楚理工学院图书馆2兆瓦光伏发电、京山年产20万件LED绿色节能灯具等项目建设，力争全年实施循环经济项目120个，完成投资110亿元。积极推广省工节本降耗、种养结合、农产品加工产业链耦合、农村生态家园等循环农业模式，促进农业资源的再循环、再利用、再增值。大力倡导绿色消费，推广高效绿色照明产品7万只，完成1000个废弃电池回收箱和164个电子废弃物回收超市规划建设，逐步向低碳社会转型。

　　强化节能减排。继续实行节能减排目标责任制管理，严格实施新上项目用能评估和环境评估审核制度，切实抓好“九小”专项治理，坚决淘汰高能耗、高污染行业的落后生产设备和工艺装置，坚决制止低水平重复建设。积极推进工业、交通、建筑三大领域节能。严格用地规划和管理，完成全市土地利用总体规划修编和第二次土地大调查工作，加强对闲置和低效土地的利用。

　　加强环境保护。围绕创建国家环境保护模范城市，抓好重点流域、区域和行业的环境综合整治。加大漳河等66处城镇集中饮用水源的保护力度，加强汉江、长湖等重点河流生态环境的修复和治理，开展竹皮河城区段和杨树港河道综合整治，严禁水库投肥养殖。完成杨树港、沙洋和后港污水处理厂建设，建成李宁（荆门）产业园污水提升泵站，逐步推广垃圾分类收集、运输和处理模式。综合治理中心城区粉尘和噪音污染。

　　积极推进国家现代林业示范市建设。加快中心城区道路绿化景观带、城郊绿化防护林带、庭院小区园林化和义务植树基地化建设。倡导社会力量参与林业碳汇事业，积极实施碳汇造林，深入开展全民义务植树和绿地捐建、共建、认养活动。支持各县市区积极创建全国绿化模范县。完成武荆高速公路荆门段、襄荆高速公路沙洋段沿线的绿化工作。高标准建设京山、钟祥、屈家岭百公里生态文化旅游长廊和掇刀现代多功能林业展示长廊。力争全年完成人工造林30万亩、建设绿色通道600公里，“四旁”植树1000万株，新建花卉苗木基地3万亩。

　　（七）深化改革开放，增强发展动力和活力

　　深化各项改革。完成市、县（市、区）政府机构改革和事业单位岗位设置管理改革。稳步推进医药卫生体制改革，加快建立基本医疗保障制度，启动公立医院改革试点。实施文化体制改革，进一步解放和发展文化生产力。推进非工领域国有企业产权制度改革，完成市直部门所属经济实体主辅分离和脱钩改制，实现经营性国有资产统一监管。继续深化农村综合改革，完善村民“一事一议”筹资筹劳办法，健全农村公益事业建设机制；做好土地承包管理工作，引导农村土地有序流转；探索城中村、园中村、城郊村集体资产改革，办好1-2个改革试点。深化财政管理改革，完善公共财政预算体系，稳步推进国有资本经营预算、政府性基金预算、社会保障预算工作，逐步健全政府预算体系。坚持依法治税，加强财政收入征管，全面完成财政目标任务。强化政府投资项目预算评审，健全和完善专项资金管理制度，加强财政监督检查，努力提高财政资金使用效益。

　　加大招商引资力度。进一步营造重商、敬商、安商、扶商的良好氛围，调整招商策略，以产业招商为主线，围绕主导产业和重点企业，大力引进龙头型、基地型、产业链型等带动力强的项目，引进与支柱产业和特色产业优势互补的配套项目，力争招商引资到位资金增长20%以上。举办第23届中国中西部经济技术协作区年会、体育产品博览会，组团参加第七届湖北武汉.台湾周，积极参与东部沿海、环渤海经济圈各类招商活动，承接产业转移。

　　深入推进全民创业。落实国家和省支持民营经济发展的政策措施，对本地创业主体与外地客商一视同仁，创业项目与招商项目同等待遇，倾力提供优质服务。积极推动机制、技术和管理创新，引导民营企业走科技型、外向型和专业化、规模化、集群化的发展道路。加大企业家培训力度，培养一批新的本地企业家群体。鼓励和引导有实力、懂技术、会经营的荆门籍人士回乡创业。继续开展“精彩荆门.创业之星”等评选表彰活动，形成创业有功、致富光荣的良好氛围。

　　推动出口稳定增长。加快转变外贸增长方式，优化出口产品结构，扩大农副产品、纺织服装等优势产品出口，推动机电、矿产品、化工医药等产品出口由数量规模型向质量效益型转变。重点扶持天茂集团、中集（宏图）能源装备、潭龙石材、普安阀门、金汉江、楚玉食品、鹏昌蛋业、山缘香菇等有潜力的出口企业加快发展。组织出口企业积极参加广交会、世界原料药展等重点展会，巩固老市场、拓展新兴市场。大力实施“走出去”战略，积极开展对外工程承包和对外劳务输出。

　　（八）着力保障和改善民生，全面发展社会事业

　　促进就业再就业。深入推进创业型城市创建工作，落实税收优惠、创业指导等政策措施，加大创业资金支持力度，力争小额担保贷款突破1亿元。加强就业指导、培训和服务，积极促进高校毕业生、城镇困难群众、零就业家庭和进城农民就业。发挥好政府投资和重大项目带动就业的作用，支持中小企业和劳动密集型企业发展，确保有就业愿望而不择业的人员充分就业。

　　健全社会保障体系。加大社保扩面征缴力度，力争参保人数达到180万人次。推进城镇企业职工养老保险省级统筹，抓好城镇企业职工基本养老保险关系转移接续工作。继续实施“五缓四减三补贴”政策，落实社保、岗位和转岗补贴1200万元。抓好原城镇集体企业未参保职工和被征地农民的社会保障工作。稳妥推进居民医保门诊医疗费用统筹试点，扩大高危行业、高危人群特别是农民工工伤保险覆盖面。提高财政对新型农村合作医疗补助标准，扩大门诊统筹覆盖面，为1.3万名左右农村孕产妇实施住院分娩补助。支持钟祥实施新型农村社会养老保险试点，力争覆盖面60%以上；支持掇刀自主开展试点。全面启动城市低收入家庭认定工作，加大城乡居民最低生活保障力度。完善大病医疗救助和临时救助制度。做好五保户集中供养工作，全面启动“千福园”养老服务建设工程，稳步发展老年服务业，推进社会福利社会化。

　　发展各项社会事业。进一步推进义务教育经费保障机制改革，积极实施中小学校舍安全工程，全面落实教师绩效工资制度，促进教育均衡发展。支持荆门职教集团创建全国千所职业教育示范学校，支持荆楚理工学院的建设和发展。贯彻实施《全民科技素质行动计划纲要》。大力发展文化产业，做大做强荆门日报传媒集团。提升公共文化服务水平，改扩建一批乡镇综合文化站。加强文物和非物质文化遗产保护，推进引江济汉考古工程。完善公共卫生服务体系，抓好甲型H1N1流感、艾滋病、血吸虫病、结核病等疾病防治。严格落实人口与计划生育政策，加强流动人口计划生育服务管理，开展计划生育“三级联创”，做好第六次人口普查工作。推进社区“三基”行动计划，深化和谐社区创建活动，提高社区管理和服务水平。积极支持驻荆部队建设，抓好国防动员、人民防空和民兵预备役工作。广泛开展国防教育。认真落实军人及军属优抚安置政策，做好军队转业干部、复员退伍军人接收安置工作。深入开展双拥共建活动，增强军政军民团结。继续做好对口援疆工作。扎实推进第二届地方志编修工作，进一步完善住房公积金制度，关心支持工会、共青团、工商联、妇女、儿童工作，做好通信、邮政、烟草、盐业、成品油工作，积极推进民族宗教、广播电影电视、新闻出版、老龄、残疾人、档案、外事侨务、台湾事务、仲裁、气象、防震、减灾等社会事业发展。

　　构建平安荆门。加强法制宣传和法律援助工作，全面落实“五五”普法任务，深入推进法治荆门建设。严厉打击违法犯罪活动，推进治安防范综合治理社会化。加快治安视频监控系统建设工程，确保中心城区主干道实现全覆盖。完善应急管理工作机制，提高政府维护稳定和处置突发性事件的能力。落实安全生产责任制，抓好产品质量、食品、药品和交通安全监管，坚决防止和遏制重特大事故发生。坚持开展领导干部“四访促和谐”主题活动，抓好群众信访接待中心建设，加大积案化解和依法处置力度。进一步健全人民调解、行政调解、司法调解相衔接的大调解工作机制，妥善处理人民群众合理诉求。

　　各位代表，今年，我们将继续为群众办好10件实事：

　　1、城镇新增就业4.1万人，扶持自主创业4000人，带动就业1.6万人；培训农村技能人员8000人。

　　2、资助城乡低保对象、农村五保对象分别参加城镇居民基本医疗保险和新农合；实施“大病关爱行动”，大病救助人数达到1万人次以上，救助资金1500万元以上。

　　3、建成廉租住房2210套，新增廉租住房租赁补贴6215户，对低收入住房困难家庭做到应保尽保。

　　4、加强中心城区空气环境监管和污染治理，确保全年达到优良天数保持在90％以上；改善中心城区噪声环境质量。

　　5、新增中心城区公共停车车位500个；新建公厕10座；筹资400万元对20条小街小巷进行改造，配套环卫、绿化、路灯、排水等设施。

　　6、完成30万人的农村饮水安全工程建设，为2万个农户配备彩钢板组合“丰产仓”2万套。

　　7、新建通村公路941公里，新建杨竹流域示范区公路24公里，新建通组公路路基2000公里、碎石路面1000公里；新建农村公路候车棚100个、招呼站200个。

　　8、发展村级综合服务社200个；资助建设40个村级文化活动中心和文化中心户，建设80个农民体育健身工程。新建和改造标准农家店200家。

　　9、加大扶贫助学力度，资助100名应届荆门籍贫困大学生入学；对中等职业学校1万名贫困学生进行资助，免费为1800名城市贫困学生提供教科书；免除市特殊教育学校在校学生杂费、课本费和生活费。

　　10、保障弱势群体的权益，为120名0-6岁贫困残疾儿童实施抢救性康复救助，为困难群众和农民工提供法律援助。

　　三、切实加强政府自身建设

　　新的形势和任务对政府工作提出了新的更高要求。各级政府要深入学习实践科学发展观，进一步解放思想，切实转变政府职能和工作作风，真正做到对人民负责、受人民监督、让人民满意。

　　（一）提高能力，增强推动发展的创造性。继续推进政府执行力和能力建设，增强“能力席位”意识，着力提高政府工作人员谋划发展、统筹协调、公共服务、社会管理、维护稳定的本领。更加注重提高学习能力，按照博学、勤思、力行的要求，扎实推进学习型政府建设，做到学以增智、学以立德、学以致用，不断增强贯彻落实科学发展观的自觉性和坚定性。更加注重提高谋划能力，解放思想，创新观念，深入研究事关荆门长远发展的全局性、战略性、关键性问题，勇于破除制约发展的制度障碍，善于破解阻碍发展的各种难题。更加注重提高操作能力，把长远规划与具体措施结合起来，将思路战略方案化、将方案谋划项目化、将项目实施具体化，一件事一件事去落实，一个项目一个项目去推进，一个问题一个问题去解决，确保各项工作落到实处。

　　（二）转变职能，增强政府工作的实效性。以政府机构改革为契机，整合部门职能，优化机构设置，健全和落实岗位责任制，完善部门协同配合机制，切实把工作重点放到服务市场主体和优化发展环境上。继续深化行政审批制度改革，减少和规范行政审批事项，简化办事程序，规范行政审批行为。不断创新工作模式和工作流程，进一步提高政府工作效能。建立健全政府新闻发布、政府网站和政府公报等制度和平台，适时公开政府确定的各项重要工作的进展情况，及时解决市民反映的热点难点问题，保障群众的知情权、参与权和监督权，打造“阳光政府”。

　　（三）依法行政，增强施政行为的规范性。始终坚持党委的正确领导，自觉接受市人大及其常委会的法律监督、工作监督和市政协的民主监督，主动听取民主党派、工商联、无党派人士和各人民团体的意见和建议。深入贯彻国务院和省政府关于加强市、县政府依法行政的决定和实施意见，坚持把依法行政作为政府工作的基本准则。进一步推进行政执法体制改革，完善行政执法责任制、评议考核制、行政执法过错和错案责任追究制，规范执法行为，促进行政机关和工作人员依法履行职责。

　　（四）加强监督，增强廉政勤政的自觉性。强化行政问责，加强电子监察建设，加大对财政资金、重大项目的审计监督力度，规范招投标秩序，从严查处工程建设、土地出让、产权交易和政府采购等领域的腐败行为。推行政府精细化管理，继续治理“小金库”，严格控制行政支出，减少会议、文件和应酬性活动，努力降低行政成本。认真学习贯彻党员领导干部廉洁从政准则，加强廉政制度建设，完善惩治和预防腐败体系，全面落实廉政建设责任制，坚决查处腐败大案要案，树立清正廉洁的政府形象。

　　编制“十二五”规划是今年的一项重要任务。我们将全面分析“十二五”时期荆门经济社会发展面临的形势，集思广益、群策群力，切实做好规划编制工作，增强规划的前瞻性、针对性和可操作性，努力使“十二五”规划成为科学指导全市经济社会发展的宏伟蓝图和行动纲领。

　　各位代表！荆门正处于经济社会加快发展的关键时期，困难和挑战考验着我们，机遇和期待召唤着我们，责任和使命激励着我们。让我们更加紧密地团结在以胡锦涛同志为总书记的党中央周围，在省委、省政府和市委的正确领导下，以只争朝夕的精神、坚韧不拔的毅力、求真务实的作风，开拓创新，扎实苦干，为推动荆门加快崛起而努力奋斗！
